# Cochlear Implant Reliability: On the Reporting of Rates of Revision Surgery

**DOI:** 10.1007/s12070-020-01795-z

**Published:** 2020-04-01

**Authors:** Graham O’Neill, Neil S. Tolley

**Affiliations:** Department of Otolaryngology, Head and Neck Surgery, St Mary’s Hospital, London, UK

**Keywords:** Implanted device, Complications, Reliability, Revision surgery, Health service

## Abstract

The aim of this study was to determine the magnitude of the risks associated with cochlear implantation. Results from a pool of thirty clinical studies involving cochlear implantation in over 6300 children were obtained from an internet search. The relevant data were transformed to a common time base (patient time) to allow an evaluation of events following implantation. The main outcome measure was cumulative survival probability for all-cause revision surgery. Over 10 years this was estimated to be 0.71. Thus, at 10 years post-implantation close to 30% of children with unilateral implants will have undergone revision surgery. This figure is considerably greater than that commonly reported for overall revision rates and illustrates the importance of interpreting results with respect to the relevant time frame. When non and low-use is incorporated into the analysis the above figure rises to about 37% of children affected. The findings raise concerns about the information provided to both individuals and regulatory bodies regarding the risks associated with cochlear implantation. The consequences for bilateral implantation are apparent. Our recommendations are i) a full disclosure to parents and children of the true magnitude of the risks and ii) for a body with significant expertise in reliability and systems engineering, and no conflicts of interest, to play a major role in the regulatory management of this service.

## Introduction

Many thousands of children have undergone cochlear implant surgery worldwide with bilateral implants being increasingly prescribed in more recent years. The intervention is often described as safe and reliable. However, rates of revision surgery are almost exclusively reported as an ‘overall’ figure which, because of the staggered-entry nature of the studies, is an incorrect and misleading metric. The accrual of patients over the course of a study masks both the magnitude and temporal characteristics of variables of interest since standard statistical measures based upon the whole group contain time-related biases. Life-table (actuarial) or Kaplan–Meier methods can be used to accommodate these effects but even here serious errors can be generated under certain conditions [1–4]. Regarding cochlear implant revision surgery, published papers contain information on both overall incidence rates and surgical details but time relevant information is, in the main, limited. Although efforts have been made to improve reporting standards, comparing results from different studies and sources is difficult and considered by some to be an almost impossible task [5, 6]. This is disconcerting because it indicates an inability to determine reliability with confidence, whether of the implant alone or of the intervention as a whole. The only study we are aware of to seriously address this problem is that of Wang et al. [7] which involved analysis of revision surgery in their own patient population. However, the effect of implant recalls because of high failure rates is not well represented. For example, the high failure rate of the Cochlear^®^ Nucleus C1500 series (released 2009, recalled 2011) is not reflected in the clinical results (their Fig. 4). Also, the recall of an implant by Advanced Bionics in 2010 was not represented since the authors report only using one device from this manufacturer. In order to obtain a wider body of evidence, we describe in this paper how results gleaned from a pool of clinical studies were transformed to a common time base to allow a better evaluation of events following cochlear implantation and hence a better evaluation of reliability.

## Methods

The study primarily concerns implantation in children. Also, the terms ‘reliability’ and ‘failure’ are not confined to hardware or technical issues but relate to any problem associated with cochlear implantation necessitating revision surgery under general anaesthesia and also to non-use—a fair description of failure, at least from the child’s point of view. The method used for analysis is to obtain the failure characteristics of each of the components separately and then combine them to obtain the failure, or unreliability, function F(t). The reliability (survival) probability R(t) is then R(t) = 1 − F(t).

### Data Search

Numerous papers and abstracts were obtained from the internet using keywords of ‘cochlear implantation’ with ‘paediatric’, ‘revision surgery’ and ‘reimplantation’. These were then filtered as described by the flowchart below to leave 30 studies suitable for analysis.
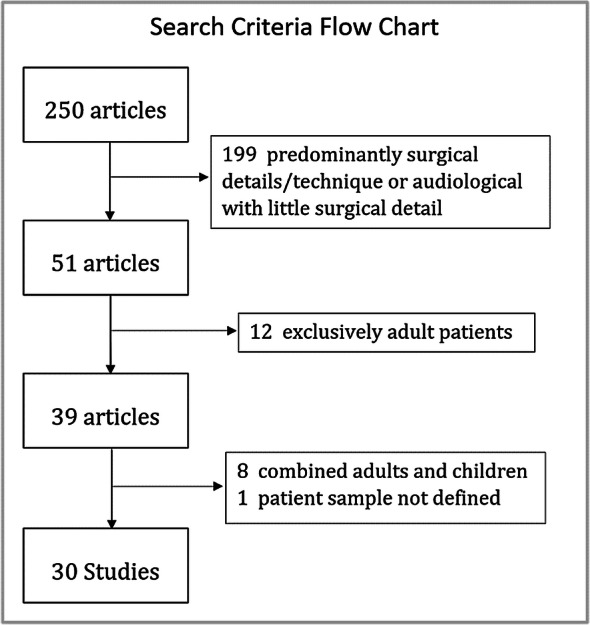


Since longer study lengths usually involve complicating factors such as increasing numbers of patients likely to be lost to follow-up, electing to have device up-grades and/or becoming non-users [8, 9], studies of over 17 years duration were excluded for the calculations described above. Out of an initial 250 articles, 199 were either predominantly surgical details/technique or audiological with little surgical detail. Of the remaining 51 articles, 12 involved exclusively adult patients, leaving 39 articles of which we obtained 37 full papers, 1 conference abstract and 1 newsletter. Of these, 8 were combined results (adults and children) and 1 did not define the patient sample. This left thirty studies, with study lengths of up to 17 years and involving over 6300 children. The earliest start date was 1984 with a study length of 11 years; the latest end date was 2016 with a study length of 14 years. No attempt was made to conduct a device specific analysis. In the allocation to specific categories, we took a conservative approach and the following assumptions made as described below:

1. Where information was reported for ‘explantation without reimplantation’, but not included in the overall results, we adjusted the numbers to include all reported explantations.
2. Where numbers for ‘explantation without reimplantation’ were not reported we assumed that total explantations was equal to the number of reimplantations.
3. In cases of post-operative infection requiring explantation, reimplantation is usually performed at a later date. That is, rates reported for reimplantation underestimate the true surgical rates relating to these cases. We were unable to adjust for this in our analysis of revision surgery where the relevant information was not provided. This also applied to multiple operations for complications not involving reimplantation.
4. Studies involving both children and adults did not state the start date for implantation in children. We were able to make an adjustment for two studies from additional information where the paediatric programme started after that for adults. For the rest we assumed that the implantation periods were the same.
5. We based our calculations on implant numbers rather than on the number of children unless only the latter was available (for studies where a proportion of children have bilateral implants, the failure rate based upon implant numbers is lower than the failure rate per child).

### Theoretical Considerations

For the purpose of this study revision surgery is categorized into surgery involving device explantation (e.g. device fault or medical/surgical complications necessitating device removal) and surgery which does not involve explantation (e.g. medical/surgical complications or device movement). This can be expressed as:

1 $$ REV_{c} \left( t \right) = EXP_{c} (t) + SUR_{c} (t) $$

where for ‘t’ years (patient time) measured from the time of implantation at time t = 0,

$$REV_{c}(t) {\text{ is the cumulative all-cause revision surgery (\%)}} = 100 \times \left(\frac{{number\,of\,revisions}}{{number\,of\,implants}}\right)$$

$$EXP_{c}(t){\text{ is the cumulative surgery involving explantation (\%)}} =100 \times \left(\frac{number\,of\,explantations}{number\,of\,implants}\right)$$

$$SUR_{c}(t) {\text { is the cumulative surgery involving everything other than explantation (\%)}}
= 100 \times \left(\frac{number\,of\,operations\,other\,than\,explantation}{number\,of\,implants}\right)$$

**Fig. 1:**
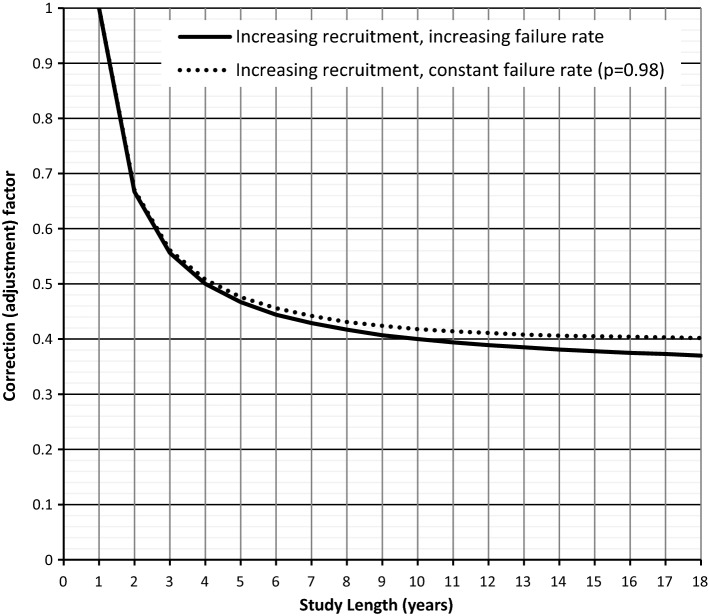
Effect of patient recruitment over the course of a study (‘staggered-entry’)—see “Appendix 1”. For example, for a 12 year implantation period results based upon the overall number of explantations need to be multiplied by a factor of 2.5 (1/0.4) to obtain explantations in patient time. That is, using simple ‘overall’ incidence figures significantly underestimates the failure rate

The extent to which the accrual of patients over the study period affects the calculation of cumulative explantation percentage is shown in Fig. 1. The analysis assumes an increasing accrual rate over the study period which seems reasonable given the information available [10–12]. Details of the construction of the graphs are given in “Appendix 1”. Results from follow-up studies were used in their original form i.e. not adjusted for ‘staggered entry’ as described above, and identified where appropriate in the presentation of results.

#### Ethical Considerations

This is a “service review” of published data relating to CI reliability. It does not involve patient study or contact. Ethical review was not necessary.

## Results

### The Failure Characteristic Relating to Explantation

**Fig. 2:**
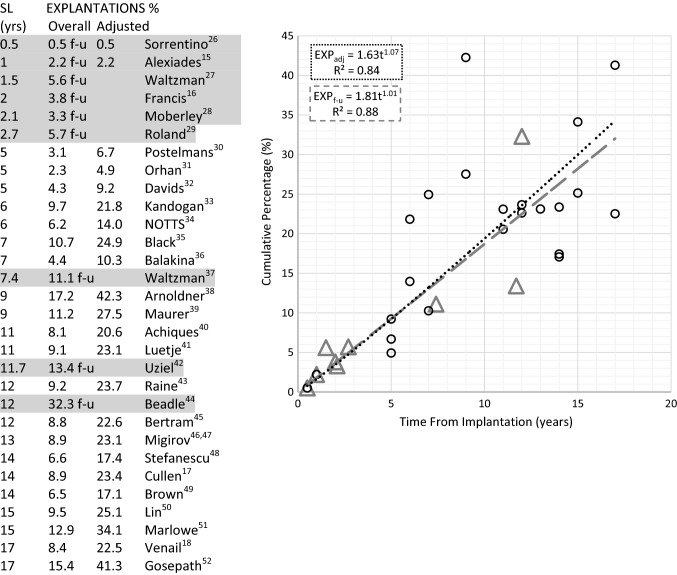
Assessment of cumulative percentage for explantations (children): Triangles: nine follow-up studies (f − u). Circles: Twenty-three studies (adj) reporting ‘overall’ figures. The latter having been adjusted for ‘staggerd-entry’. The difference between the two methods for estimation of cumulative explantations at 10 years post-implantation is less than 5% (18.5% cf 19.2%). Use of all the data gives a figure of 19.2%

Applying the study length (SL) correction factor to those clinical studies reporting overall explantation percentages gave the results shown in Fig. 2. Also shown are the results from a number of studies where explantations can be located either to a specific time frame post implantation or to an approximate follow-up period. Using all the data points gives the relationship:

$$ EXP_{c} \left( t \right) = 1.88\, \times \,t^{1.01} \left( {{\text{correlation}}\,{\text{coefficient}} = 0.84} \right) $$

The relationship is indicative of an increasing failure rate (decreasing reliability) over time. For unilateral implants, at 10 years post-implantation 19% of children will have undergone surgery involving explantation, with or without reimplantation.

### The Failure Characteristic Relating to Surgery Without Explantation

**Fig. 3:**
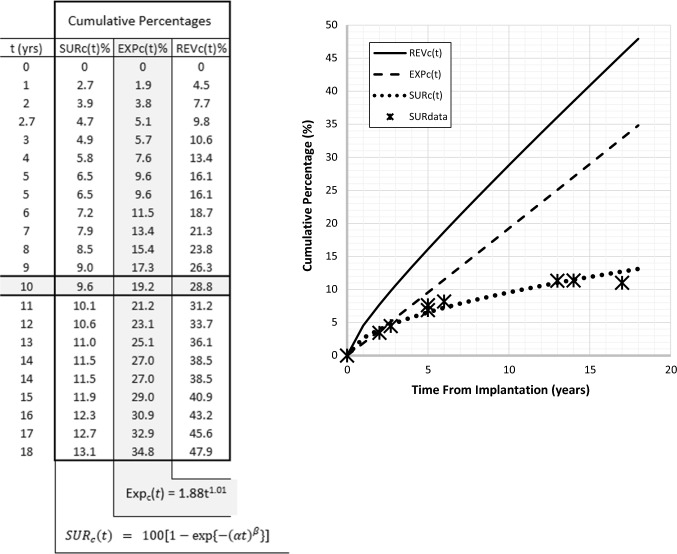
Components of cochlear implant revision surgery (children). REVc(t)—Cumulative ‘all-cause’ revision surgery = EXPc(t) + SURc(t); EXPc(t)—Cumulative surgery involving device explantation; SURc(t)—Cumulative surgery involving everything other than explantation (Weibull curve fit). At 10 years post-implantaion, ‘all-cause’ revision surgery is 29%; SURdata—Clinical data which has been transformed to cumulative values (see Table 1)

This was determined by first considering the contribution of explantation to overall revision for different study lengths. Explantations make up about 50% of revision surgery over the short term increasing to about 80% over the long term. From Eq. 1, given that

$$ SUR_{c} (t) = EXP_{c} (t)\left[ {\left\{ {\frac{{EXP_{c} (t)}}{{REV_{c} (t)}}} \right\}^{ - 1} - 1} \right] $$

cumulative surgery without device explantation was estimated from data at several time periods. The relevant data and calculations are shown in Table 1. The corresponding two-parameter Weibull curve fit, shown in Fig. 3, is of the form:-

$$ SUR_{c} \left( t \right)  =  100\left[ {1 - \exp \left\{ { - \left( {\alpha t} \right)^{\beta } } \right\}} \right] \quad  where\quad \alpha = 1.78 \times 10^{ - 3} \,{\text{and}}\,\beta = 0.57 $$

In contrast to surgery involving device explantation this reflects a decreasing failure rate with respect to time (β < 1). Using the same example as above, at 10 years post-implantation about 10% of children will have undergone surgery without device explantation.

**Table 1:**
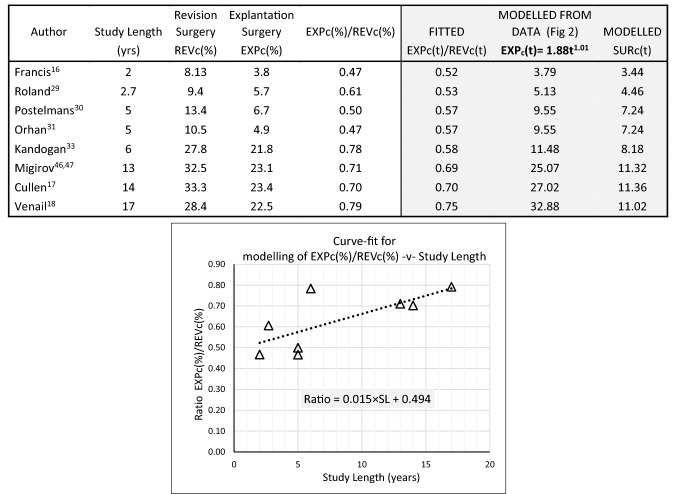
Cochlear implants (children)—estimation of revision surgery which does not involve device explantation SURc(t)

The ratio of EXP_c_(%)/REV_c_(%) was evaluated for each of the studies above. The relationship between this ratio and study period was obtained by a curve-fit as shown in the graph. The resulting linear equation was then used to model EXP_c_(t)/REV_c_(t) (fitted) at the respective study lengths. These fitted values were then used in the equation: SUR_c_(t) = EXP_c_(t) [{EXP_c_(t)/REV_c_(t)}^−1^ − 1] (see text), to provide a modelled estimate for SUR_c_(t)

### The Failure Characteristic Relating to (All-Cause) Revision Surgery

Figure 3 shows cumulative revision surgery obtained from the analyses above. At 10 years post-implantation close to 30% of children with unilateral implants will have undergone revision surgery. Clearly, this figure is considerably greater than that commonly reported for overall revision rates and illustrates the importance of interpreting results with respect to the relevant time frame.

### Non-Use

From Raine et al. [13] and Contrera et al. [14] we modeled the better results (lower non-use) of the latter and used a failure rate of 1.2% year^−1^. Over 10 years the cumulative survival probability is (1 − 0.012 × 10) = 0.88. That is, if the failure rate is f_r_ (= 0.012), then the cumulative survival probability R(t)_n−u_ = (1 − f_r_ × t).

### Reliability Curve

**Fig. 4:**
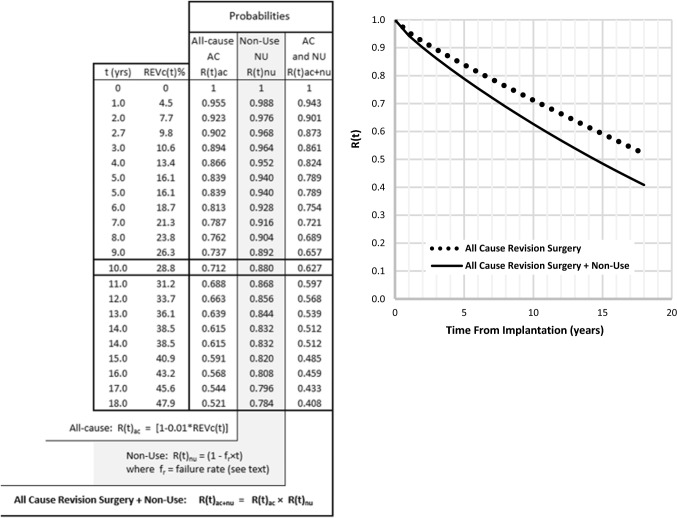
Unilateral cochlear implant reliability curve. At 10 years post-implantation: R(t) for all-cause revision surgery is approx. 0.7. Since failure (un-reliability) F(t) = 1 − R(t) = 0.3, then 30% of original patients, implanted at time t = 0, have experienced failure. R(t) for all-cause revision surgery + non-use is 0.63. i.e. F(t) = 0.37 or 37% of original patients have experienced failure involving revision surgery and/or non-use

The components described above can be combined to provide a conventional reliability (survival) curve. Using the term F(t) to represent the failure, or unreliability, function in the form of REV_c_(t) in Eq. 1, but without being expressed as a percentage then, for values of REV_c_(t) between 0 and 100%, F(t) will have corresponding values between 0 and 1.0. The reliability (survival) probability R(t) is then R(t) = 1 − F(t). In Fig. 4, R(t) is plotted vertically and takes on values between 1.0 (100% reliability at time t = 0, i.e. no failures) and 0 (all cases have experienced failure). The abscissa is patient time in years. When incorporating non-use into this analysis we multiplied reliability (survival) probabilities on the evidence that some non-users would also have undergone explantation.

### Device Failure Rate

Clinical papers report, on average, that device failures constitute about 80% of explantations [15–18], giving a failure of about 15% over 10 years of implant use (cumulative survival probability = 0.85). This contrasts dramatically with figures quoted by manufacturers. A point well made by Roby et al. [19].

## Discussion

Cochlear implantation is described in the literature as a safe and reliable intervention for severe-to-profound bilateral sensorineural hearing loss. In particular, descriptions refer to low overall revision rates (often with no reference to a time frame) and to device reliability with survival percentages frequently quoted in the high 90 s. The results of this present study give reason to challenge these descriptions. Firstly, regarding revision surgery, there is a very significant difference in the figures where the simple overall percentage grossly underestimates the more representative cumulative value as measured in patient time. Secondly, whereas manufacturer’s data sheets for device reliability at, say, 10 years quote cumulative survival percentages (CSP) of 94% and above (cumulative failure of ≤ 6%), clinical results indicate values in the region of 85% (cumulative failure of ≃ 15%). That is, from a clinical perspective, device failure rates appear to be about three times greater than those reported by the manufacturers. Clearly, large numbers of children undergoing explant surgery do not show up as failures. Also, non-users with implanted devices within specification are not counted as failures neither are those who undergo revision surgery without device explantation. Thus, current figures for surgery and for device reliability distort the magnitude of risk. We contend that the most relevant information for parents and patients is the cumulative all-cause revision surgery (%) over, typically, 10 years. Given that this figure is close to 30% for unilateral implants the consequences for bilateral implantation are clear. We believe that non-use should also be considered a failure since the implant is, in essence, ineffective compared to its intended purpose. Expanding upon some of the issues described above:-

### Non-reporting of Numbers of ‘Explantation Without Reimplantation’

Either for medical reasons or by choice, some patients undergo explantation without reimplantation. In the few studies (four) which contained the relevant information, we found that relying upon the reported number of reimplantations underestimated the total number of explantations by about 10% (median 10.3%, range 6.7–11.1%).

### Patients ‘Lost-to-Follow-Up’ (LTFU)

Of the original numbers in the Contrera et al. [14] study, at 10 years post-implantation, 25% were either ‘lost-to-follow-up’ (15%) or non or not regular users (10%). Taking a value mid-way between the best and worst case scenarios (no/all LTFU being non or low users) provides an estimate of 18% of the original patients being non or low users at 10 years following implantation. Also, it is clear from this study that those ‘lost-to-follow-up’ and those who are ‘known non-users’ are not random subsets of the total group. Not taking account of this inevitably results in an underestimate of the failure rate. Our suggestion of incorporating non-use (1.2% year^−1^—see ‘Results’ above) with all-cause revision surgery is more accurately a metric of ‘failure’.

### Results from Long Study Periods

Long study periods are by default more recent publications. It is evident that higher numbers of both children and children receiving bilateral implants occur in the later study period [20–22] (only unilateral implants were provided up to the early 2000s). With this ‘implants -v- time’ profile the results of such studies will be biased by the large number of newer implants with a short duration of use even when total study length is 20 years or more. The combined effects of this with significant ‘lost-to-follow-up’ numbers, as described above can result in erroneous failure rates being quoted.

### A Simple Check

During the earlier years of cochlear implantation a reasonable ‘first approximation’ of reliability could have been carried out by assuming a constant implantation rate per year for the study period and multiplying the overall revision rate by 2 (average duration of use = half the study length). Indeed, Fig. 2 includes eight studies, all published before the NICE [10] submission, where this simple check (for explantations) results in a mean of 2.4% year^−1^ (median = 2.3% year^−1^). There are also four follow-up studies giving mean and median values of 2.5% year^−1^. This contrasts with an annual re-implantation rate of 0.6% quoted in support of implantation (NICE [10-p31]).

#### Cochlear Implants and Unilateral Hearing Loss

Being aware that implantation is also being promoted for unilateral/asymmetrical hearing loss, we would agree with The American Speech-Language-Hearing Association [23]—‘*A cochlear implant is not an option for children with Unilateral Hearing Loss. This device, which is placed surgically in the inner ear, is only for children with severe or profound hearing loss in both ears*’. This contrasts sharply with information leaflets which describe the possibility of implants providing help for single-sided deafness in a variety of situations, including the classroom [24, 25]. Given the information provided in this paper we see no sound basis for the promotion of implants for single-sided deafness.

#### Strengths and Limitations of this Study

The main strength of the study is that of results from a relatively large number of children (> 6300) combined with an appropriate measurement statistic for staggered-entry design. The main limitation relates to the data available for the analysis in that some studies were more comprehensive and/or specific than others on the reasons for revision surgery. Therefore, and in common with other branches of medicine, there is a high likelihood of under-reporting in several of the studies.

## Effect of Patient Recruitment Throughout a Study (‘Staggered-Entry’)

### Constant Failure Rate

For increasing recruitment numbers per year, let n_i_ = i × k where k = constant.

Also, for a constant failure rate, conditional probability p = constant.

$$ \begin{aligned} & {\text{Total}}\,{\text{number}}\,{\text{of}}\,{\text{explants}}\,{\text{for}}\,{\text{say}}\,{\text{a}}\,{\text{three\,year}}\,{\text{study}}\,{\text{length}}\left( {{\text{SL}} = 3} \right) = {\text{ n}}_{1} \left( {1 - {\text{p}}^{3} } \right) + {\text{n}}_{2} \left( {1 - {\text{p}}^{2} } \right) + {\text{n}}_{3} \left( {1 - {\text{p}}^{1} } \right) \\ & {\text{Total}}\,{\text{number}}\,{\text{of}}\,{\text{implants}} = {\text{n}}_{1} + {\text{n}}_{2} + {\text{n}}_{3} = 1{\text{k}} + 2{\text{k}} + 3{\text{k}} = {\text{k}} \times {\text{L}} \\ & where\, {\text{L}} = \mathop \sum \limits_{z = 1}^{SL} \left( {\text{z}} \right) \\ \end{aligned} $$

Thus,

$$ {\text{EXP}}_{c} \left( 3 \right) = \frac{{\left\{ {1\left( {1 - {\text{p}}^{3} } \right) + 2\left( {1 - {\text{p}}^{2} } \right) + 3\left( {1 - {\text{p}}^{1} } \right)} \right\}}}{\text{L}} \times 100 $$

$$ In \, general,\quad EXP_{c} \left( {\text{SL}} \right)  =  \frac{100}{{ {\text{L}}}}\mathop \sum \limits_{i = 1,j = SL}^{SL,1} {\text{i }}\left( {1 - p^{j} } \right) $$

$$ {\text{For}}\,{\text{all}}\,{\text{implantations}}\,{\text{at}}\,{\text{t}} = 0,\quad {\text{EXP}}_{\text{c}} \left( {\text{SL}} \right) = 100 \times \left( {1{-}{\text{p}}^{\text{SL}} } \right) $$

$$ Thus, the \, ratio  \frac{successive\, implantation}{all\, implantations\, at\, time\, t = 0}  =  \frac{1}{{\left( {1 - p^{SL} } \right) {\text{L}}}}\mathop \sum \limits_{i = 1,j = SL}^{SL,1} {\text{i }}\left( {1 - p^{j} } \right). $$

### Increasing Failure Rate

For increasing recruitment numbers per year, let n_i_ = i × k where k = constant.

If number of explantations per year for each n_i_ is the same = C × n_i_ where C is a constant.

$$ \begin{aligned} & {\text{Total}}\,{\text{number}}\,{\text{of}}\,{\text{explants}}\,{\text{for}}\,{\text{say}}\,{\text{a}}\,{\text{three}}\,{\text{year}}\,{\text{study}}\,{\text{length}}\left( {{\text{SL}} = 3} \right) = {\text{C}} \times {\text{n}}_{1} \times 3 + {\text{C}} \times {\text{n}}_{2} \times 2 + {\text{C}} \times {\text{n}}_{3} \times 1 = {\text{C}} . {\text{k}}\left( {1 \times 3 \, + \, 2 \times 2 \, + \, 3 \times 1} \right) \\ & {\text{Total}}\,{\text{number}}\,{\text{of}}\,{\text{implants}} = {\text{n}}_{1} + {\text{n}}_{2} + {\text{n}}_{3} = 1{\text{k}} + 2{\text{k}} + 3{\text{k}} = {\text{k}} \times {\text{L}} \\ & where\quad {\text{L}} = \mathop \sum \limits_{z = 1}^{SL} \left( {\text{z}} \right) \\ \end{aligned} $$

$$ In \, \,general,\quad EXP_{c} \left( {\text{SL}} \right)  =  \frac{{100 \times {\text{C}}}}{{ {\text{L}}}}\mathop \sum \limits_{i = 1,j = SL}^{SL,1} {\text{i }} \times {\text{j}} $$

$$ {\text{For}}\,{\text{all}}\,{\text{implantations}}\,{\text{at}}\,{\text{t}} = 0,\quad {\text{EXP}}_{\text{c}} \left( {\text{SL}} \right) = 100 \times {\text{C}} \times {\text{SL}} $$

$$ Thus,\, the\, ratio  \frac{successive\, implantation}{all\, implantations\, at\, time\, t = 0}  =  \frac{1}{{{\text{SL}} \times {\text{L}}}}\mathop \sum \limits_{i = 1,j = SL}^{SL,1} {\text{i}} \times {\text{j}}. $$

This ratio, for study lengths ranging from 1 to 18 years, is shown graphically in Fig. 1.
